# Biomarkers for Refractory Lupus Nephritis: A Microarray Study of Kidney Tissue

**DOI:** 10.3390/ijms160614276

**Published:** 2015-06-23

**Authors:** Thitima Benjachat, Pumipat Tongyoo, Pornpen Tantivitayakul, Poorichaya Somparn, Nattiya Hirankarn, Santitham Prom-On, Prapaporn Pisitkun, Asada Leelahavanichkul, Yingyos Avihingsanon, Natavudh Townamchai

**Affiliations:** 1 Biomedical Science, Interdisciplinary Program, Graduate School, Chulalongkorn University, Bangkok 10330, Thailand; E-Mails: tbenjachat@gmail.com (T.B.); ptongyoo@gmail.com (P.T.); 2 Center of Excellence in Immunology and Immune-mediated Diseases, Faculty of Medicine, Chulalongkorn University, Bangkok 10330, Thailand; E-Mails: nattiyap@gmail.com (N.H.); a_leelahavanit@yahoo.com (A.L.); 3 Department of Oral Microbiology, Faculty of Dentistry, Mahidol University, Bangkok 10400, Thailand; E-Mail: phornpen_071@hotmail.com; 4 Research Affairs, Faculty of Medicine, Chulalongkorn University, Bangkok 10330, Thailand; E-Mail: pook_bio@yahoo.com; 5 Division of Immunology, Department of Microbiology, Faculty of Medicine, Chulalongkorn University, Bangkok 10330, Thailand; 6 Department of Computer Engineering, Faculty of Engineering, King Mongkut’s University of Technology Thonburi, Bangkok 10140, Thailand; E-Mail: san.promon@gmail.com; 7 Division of Allergy, Immunology, and Rheumatology, Department of Medicine, Faculty of Medicine, Ramathibodi Hospital, Mahidol University, Bangkok 10400, Thailand; E-Mail: beepisitkun@gmail.com; 8 Division of Nephrology, Department of Medicine, Faculty of Medicine, Chulalongkorn University and King Chulalongkorn Memorial Hospital, Bangkok 10330, Thailand; E-Mail: ntownamchai@gmail.com

**Keywords:** lupus nephritis, biomarker, microarrays, gene expression, chronic kidney disease

## Abstract

The prognosis of severe lupus nephritis (LN) is very different among individual patients. None of the current biomarkers can be used to predict the development of refractory LN. Because kidney histology is the gold standard for diagnosing LN, the authors hypothesize that molecular signatures detected in kidney biopsy tissue may have predictive value in determining the therapeutic response. Sixty-seven patients with biopsy-proven severely active LN by International Society of Nephrology/Renal Pathology Society (ISN/RPS) classification III/IV were recruited. Twenty-three kidney tissue samples were used for RNA microarray analysis, while the remaining 44 samples were used for validation by real-time polymerase chain reaction (PCR) gene expression analysis. From hundreds of differential gene expressions in refractory LN, 12 candidates were selected for validation based on gene expression levels as well as relevant functions. The candidate biomarkers were members of the innate immune response molecules, adhesion molecules, calcium-binding receptors, and paracellular tight junction proteins. *S100A8*, *ANXA13*, *CLDN19* and *FAM46B* were identified as the best kidney biomarkers for refractory LN, and *COL8A1* was identified as the best marker for early loss of kidney function. These new molecular markers can be used to predict refractory LN and may eventually lead to novel molecular targets for therapy.

## 1. Introduction

Throughout the years, treatment for lupus nephritis (LN) has improved. However, approximately 20% of patients with severe proliferative LN will develop refractory LN [1]. Not only do these patients fail to respond to the standard treatment, they also have a worse prognosis. In a long-term cohort of non-responder patients, only half of the patients survived and more than 80% of them progressed to end-stage kidney disease [2]. Consequently, diagnosis and prognosis of refractory LN are extremely important. Factors associated with treatment failure are non-Caucasian ethnicity, ISN/RPS class III/IV type of renal pathology, and treatment non-compliance [3]. However, there are no specific renal pathology characteristics that can accurately predict the therapeutic response for patients with ISN/RPS class III/IV LN [4]. The authors hypothesize that cellular or molecular signals at the time of active LN may contribute to the mechanisms of the disease [5,6]. Therefore, it is possible that the molecular signatures obtained from kidney tissue may be able to provide some diagnostic and prognostic values for refractory LN as well as future novel therapeutic targets [7,8,9].

A few promising biomarkers to predict the therapeutic response of LN patients have been reported. These previous studies used various sample types. A study of urine samples from active LN patients revealed that increased levels of urinary mRNA, *IP-10*, *CXCR3*, *TGF-β* and *VEGF* were associated with treatment failure [10]. Furthermore, the urinary sediment in active LN showed a high number of CD4 T-cells, supporting a role for T-helper 1 cells in this condition. Immunosuppressive treatment can reduce CD4 T-cells [11]. A study of serum samples indicated that high levels of serum IL-17 and IL-23 were associated with unfavorable response in inactive LN with immunosuppressive treatment [12]. A recent study determined a significantly increased level of large intergenic noncoding RNA expression (*linc0949*) in the peripheral blood of LN patients after treatment [13]. These biomarkers have been investigated based on known molecular functions. With the robust technology for genome-wide studies, RNA microarrays can be an unbiased way of biomarker discovery in active kidney disease of LN [14,15]. The authors believe that the molecular information from kidney tissue should be able to help in diagnosing and prognosing refractory LN. Aside from that, such biomarkers may prove to be useful in future targeted therapies and provide insightful information on factors contributing to the mechanisms of the disease.

## 2. Results and Discussion

### 2.1. Patients and Samples

Sixty-seven systemic lupus erythematosus (SLE) patients with biopsy-proven ISN/RPS class III/IV LN were studied. A kidney biopsy was performed at the time of active kidney disease. All patients had biopsy-proven class III/IV LN. Two sets of kidney samples were studied: a microarray training set (*n* = 23) and a real-time PCR validation set (*n* = 44). The characteristics of the patients from the training set and validation set are shown in Table 1 and Table 2, respectively. In this study, the patients were initially treated with an immunosuppressive regimen composed of steroids and either a cyclophosphamide or mycophenolate regimen for six months. Response to therapy was defined after six months of initial treatment (see details in the Experimental Section).

According to the protocol, all patients in the cohort were maintained on immunosuppressive drugs for at least three years. Early loss of kidney function was defined as an estimated glomerular filtration rate (GFR) below 15 mL/min, or dialysis or kidney transplantation within 12 months.

### 2.2. Intra-Renal Gene Expression in Lupus Nephritis (LN) Patients Who Did Not Respond to Treatment

This study identified certain gene expressions that can be used to diagnose and prognose refractory LN. A poor prognosis indicated that the patient did not respond to treatment. The intra-renal gene expression was measured during an active episode of LN. The molecular signatures obtained provided additional information that could not be detected by routine histology. In this cohort, the patients were divided into two groups based on the criteria described in the Methods sections: a responder group and a non-responder group. All patients had biopsy-proven LN with ISN/RPS class III or IV. It should be noted that none of the clinical parameters could predict the therapeutic responses (Table 1 and Table 2). A histological parameter, the chronicity index, was associated with the therapeutic outcome in the training group (*p* = 0.03), but not in the validation group (*p* = 0.37).

**Table 1:** Characteristics of the patients in the training set.

Characteristics	R ^a^	NR ^b^	*p*-Value
Number	14	9	n/a
Sex (F/M)	13/1	9/0	n/a
Age (years)	32.31 ± 2.62	31.06 ± 2.70	0.88
Clinical parameters			
Serum creatinine (mg/dL)	1.21 ± 0.32	1.56 ± 0.18	0.08
Proteinuria (g/day)	4.36 ± 0.80	4.33 ± 1.30	0.78
Urinary erythrocyte count (per high-power field)	92.86 ± 39.20	58.22 ± 31.82	0.37
MDRD ^c^ GFR (mL/min)	83.44 ± 11.63	51.18 ± 8.67	0.18
Renal histology			
Class III	1	1	n/a
Class IV	13	8	n/a
Activity index	10.45 ± 0.86	11.63 ± 1.84	0.55
Chronicity index	1.55 ± 0.43	3.88 ± 0.83	0.03
Steroid dose (mg/day)	30.0 ± 8.66	21.60 ± 12.02	0.83
Loss of renal function within 1 year ^d^	2 (14.29%)	5 (55.55%)	0.04

^a^ R = LN responder patients; ^b^ NR = LN non-responder patients; ^c^ MDRD = Modification of Diet in Renal Disease; ^d^ Loss of renal function = Estimated GFR below 15 mL/min or patients who have had dialysis or kidney transplantation; n/a = not applicable.

**Table 2:** Characteristics of the patients in the validation set.

Characteristics	R ^a^	NR ^b^	*p*-Value
Number	22	22	n/a
Sex (F/M)	22/0	22/0	n/a
Age (years)	34.09 ± 1.98	32.04 ± 1.84	0.41
Clinical parameters			
Serum creatinine (mg/dL)	1.15 ± 0.22	1.44 ± 0.29	0.29
Proteinuria (g/day)	3.39 ± 0.37	3.99 ± 0.75	0.93
Urinary erythrocyte count (per high-power field)	61.57 ± 26.26	35.27 ± 13.42	0.32
MDRD ^c^ GFR (mL/min)	85.60 ± 8.90	70.56 ± 8.62	0.30
Renal histology			
Class III	3	5	n/a
Class IV	19	17	n/a
Activity index	9.38 ± 0.86	8.60 ± 1.21	0.40
Chronicity index	2.29 ± 0.51	3.15 ± 0.59	0.37
Steroid dose (mg/day)	25.59 ± 4.78	28.06 ± 6.50	0.70
Loss of renal function ^d^	3 (13.64%)	10 (31.82%)	0.02

^a^ R = LN responder patients; ^b^ NR = LN non-responder patients; ^c^ MDRD = Modification of Diet in Renal Disease; ^d^ Loss of renal function = Estimated GFR below 15 mL/min or patients who have had dialysis or kidney transplantation; n/a = not applicable.

**Figure 1:**
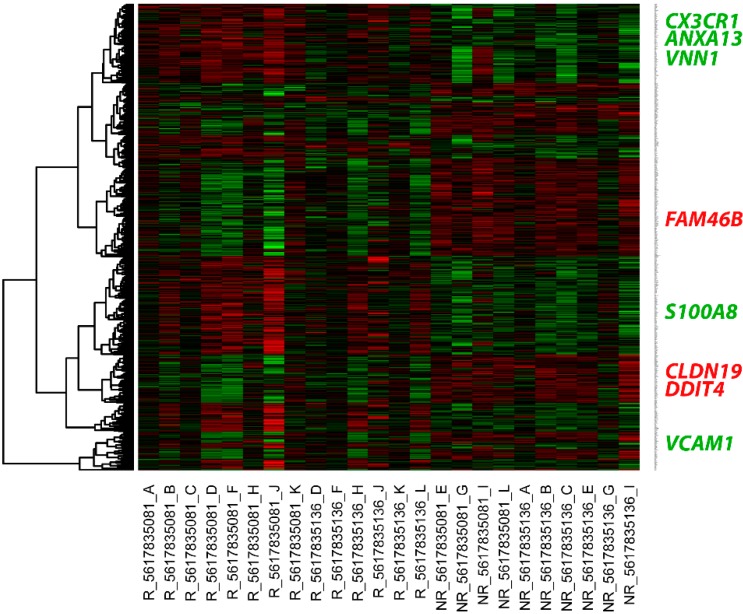
Heat map of the differential gene expressions between the lupus nephritis (LN) responder and non-responder patients. The upregulated genes are shown in red, while the downregulated genes are shown in green. Each column represents an individual kidney sample. R: LN responder patient; NR: LN non-responder patient. Gene names are indicated on the right. All validated candidate biomarkers are highlighted in green text for upregulated genes and red text for downregulated genes.

In the non-responders (*n* = 9), 353 intra-renal genes were downregulated and 396 intra-renal genes were upregulated compared with the levels in the responders (*n* = 14). A full list of the differentially expressed genes (DEGs) is shown in Supplementary Table S1. An overview of the expression pattern of DEGs by hierarchical clustering is shown in a heat map (Figure 1) with gene name labeling on the vertical axis. Genes indicated in green showed an upregulated expression pattern, while those indicated in red showed a downregulated expression pattern. Lists of the functional annotation and pathway analysis outcomes for the DEGs are shown in Supplementary Tables S2 and S3. The upregulated genes included immune response molecules, adhesion molecules, and receptors of cytokines or immunoglobulin super families. Meanwhile, the downregulated genes tended to be related to tight junctions and protein transport in the kidney.

A list of selected gene expression among patients who responded and did not respond to treatment is shown in Table 3 (change > 2-fold and *p* < 0.01). The upregulated gene expressions, namely *ANAX13*, *VCAM1*, *CX3CR1*, *VNN1* and *S100A8*, labeled in green text in Figure 1, and downregulated gene expressions, namely *CLDN19*, *DDIT4* and *FAM46B*, labeled in red text in Figure 1, were validated by real-time PCR.

**Figure 2:**
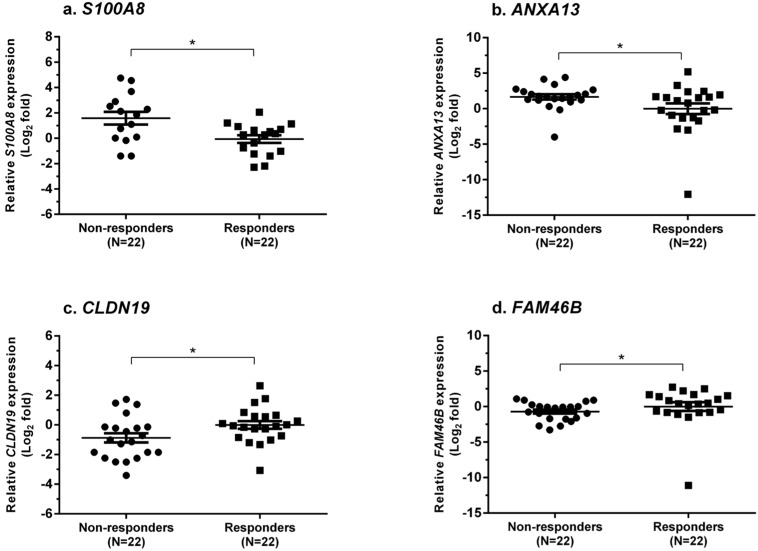
Dot-plots showing the relative gene expression levels between the LN responder and non-responder groups. The selected genes showing upregulation of gene expression were (**a**) *S100A8* and (**b**) *ANXA13*, while those showing downregulation of gene expression were (**c**) *CLDN19* and (**d**) *FAM46B*. * *p* < 0.05.

From the validation set (22 from the responder group; 22 from the non-responder group), *S100A8*, *ANXA13*, *CLDN19* and *FAM46B* were identified as biomarkers for predicting treatment failure (Figure 2). The expressions of *S100A8* and *ANXA13* were increased (1.58 ± 0.50 *vs.* −0.07 ± 0.30, log_2_ fold, *p* = 0.02 and 1.65 ± 0.37 *vs.* −0.006 ± 0.75, log_2_ fold, *p* = 0.04, respectively), whereas *CLDN19* and *FAM46B* were decreased (−0.87 ± 0.31 *vs.* −0.005 ± 0.26, log_2_ fold, *p* = 0.03 and −0.71 ± 0.28 *vs.* −0.005 ± 0.62; log_2_ fold, *p* = 0.02, respectively) in the non-responder group compared with the responder group. All validated gene expressions can be found in Supplementary Table S5. Among these markers, *S100A8* provided the best values of sensitivity and specificity for predicting resistance to treatment.

**Table 3:** List of candidate genes obtained from the therapeutic responders.

Illumina IDs	Target ID	Fold Change	*p*-Value	Gene Function or Annotation
***Upregulated genes***				
ILMN_19368 ^a^	*ANAX13*	2.22	0.0004	annexin A13
ILMN_627 ^a^	*ANAX13*	2.46	0.0011	annexin A13
ILMN_3875 ^b^	*VCAM1*	2.47	0.0022	vascular cell adhesion molecule 1
ILMN_26453 ^b^	*VCAM1*	2.38	0.0026	vascular cell adhesion molecule 1
ILMN_8593	*CX3CR1*	2.01	0.0051	chemokine (C-X3-C motif) receptor 1
ILMN_14011	*VNN1*	2.38	0.0062	vanin 1
ILMN_13072	*S100A8*	2.55	0.0095	S100 calcium-binding protein A8
***Downregulated genes***				
ILMN_6731	*CLDN19*	−2.23	0.0073	claudin 19
ILMN_13176	*DDIT4*	−2.27	0.0005	DNA-damage-inducible transcript 4
ILMN_9808	*FAM46B*	−2.70	0.0015	family with sequence similarity 46, member B

^a^ Probe ID for annexin A13; ^b^ Probe ID for vascular cell adhesion molecule 1.

### 2.3. Intra-Renal Gene Expression in LN Patients Who Had Loss of Kidney Function within 12 Months

The secondary objective of this study was to identify the differences in gene expression between patients with or without loss of kidney function within 12 months. As expected, there were more patients in the non-responder group who lost their kidney function (Table 1 and Table 2). Even though the patients were treated with the standard immunosuppressive drugs, they still developed end-stage kidney disease within 12 months. It should be noted that no specific histological characteristics could predict the loss of kidney function. The activity index did not differ between the two groups. Even though the chronicity index was quite high in the group of patients who lost their kidney function, this was not significant in the validation sample set.

**Figure 3:**
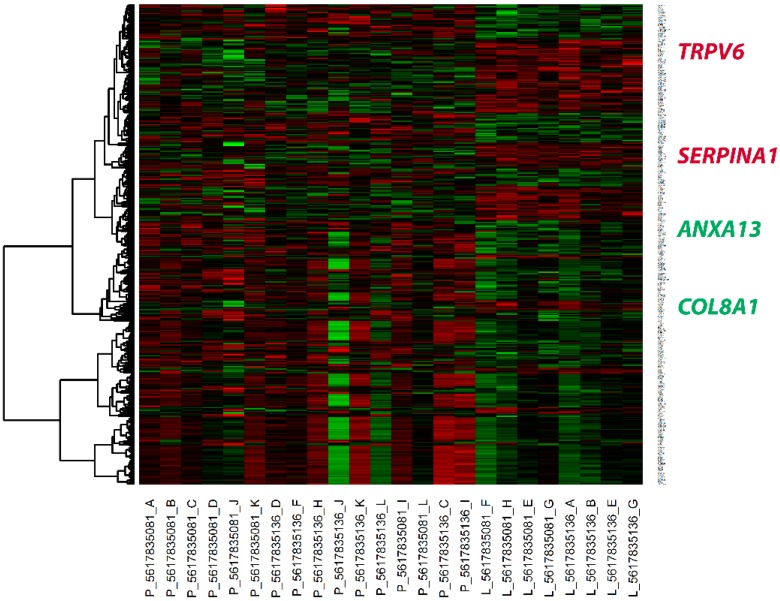
Heat map of differential gene expression between LN patients with or without loss of kidney function. The upregulated genes are shown in red, while the downregulated genes are shown in green. Each column represents an individual kidney sample. P: preserved kidney function; L: loss of kidney function. Gene names are indicated on the right. All validated candidate biomarkers are highlighted in green text for upregulated genes and red text for downregulated genes.

From the intra-renal gene expression, 13 patients lost their kidney function while 31 patients preserved their kidney function. Using the data from the 13 patients who lost their kidney function, 133 genes were downregulated and 253 genes were upregulated. The full list of DEGs is shown in Supplementary Table S4. The expression pattern of DEGs in patients with and without early loss of kidney function is shown in Figure 3. Lists of the functional annotation and pathway analysis outcomes are shown in Supplementary Tables S2 and S3. The biological relevance to the loss of kidney function revealed upregulated function of extracellular matrix structure proteins. In contrast, patients who preserved their kidney function tended to overexpress cytokine and signaling molecules. Four genes with high fold changes and disease-related biological functions were selected. These genes, namely *ANXA13*, *COL8A1*, *SERPINA1* and *TRPV6*, were selected for validation by real-time PCR (Table 4).

**Table 4:** List of candidate genes from patients with early loss of renal function.

Illumina IDs	Target ID	Fold Change	*p*-Value	Gene Function or Annotation
***Upregulated genes***				
ILMN_19368	*ANAX13*	1.82	0.03	annexin A13
ILMN_10408	*COL8A1*	1.85	0.04	collagen, type VIII, alpha 1
***Downregulated genes***				
ILMN_1034	*SERPINA1*	−1.39	0.03	serpin peptidase inhibitor, clade A, member 1
ILMN_13176	*TRPV1*	−1.38	0.02	transient receptor potential cation channel, subfamily V, member 6

**Figure 4:**
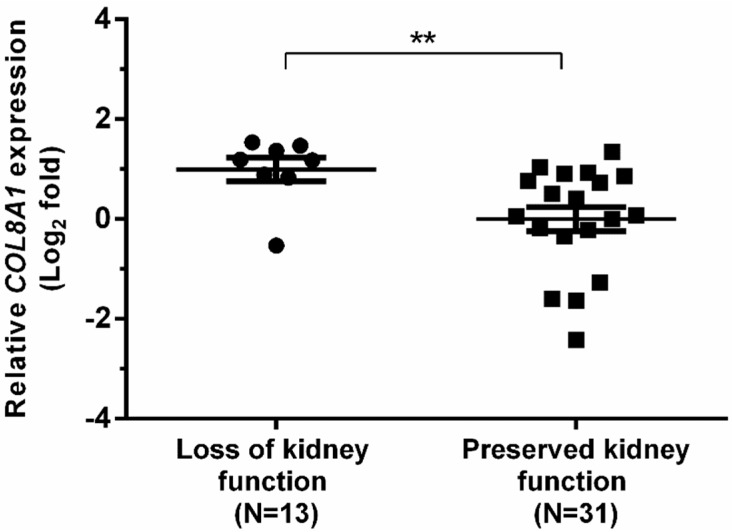
Dot-plots showing the relative gene expression levels of *COL8A1*. There was upregulation of the gene expression in the group who had loss of kidney function within 12 months. ** *p* < 0.01.

From the validated set (31 from patients with preserved kidney function; 13 from patients who lost their kidney function), the *COL8A1* gene was proven to be a biomarker that could predict the loss of kidney function within 12 months. From a receiver-operating curve analysis, *COL8A1* could predict loss of kidney function with a sensitivity of 87.5% and specificity of 73.7%. Patients who lost their kidney function had higher expression of *COL8A1* compared with those whose kidney function was preserved (0.99 ± 0.24 *vs.* −0.004 ± 0.24, log_2_ fold, *p* = 0.007, Figure 4). All validated gene expressions can be found in Supplementary Table S6.

### 2.4. Discussion

Resistance to the standard treatment contributes to the development of refractory LN, which can rapidly result in end-stage kidney disease. Many patients with severe forms of LN will eventually develop refractory LN, which is often unpredictable in nature. This study discovered 749 genes as novel molecular signatures of refractory LN using the unbiased molecular approach of RNA microarrays. The involvement of biomarkers in the mechanism of the disease is beyond the scope of the present study. By using this reliable validation method, a number of novel biomarkers were observed. There was increased expression of innate immune response molecules, adhesion molecules, collagens, and calcium-binding receptors in the kidney tissue of patients with refractory LN. Conversely, the expressions of paracellular tight junction molecules were decreased.

The majority of previous studies looked at the differences between LN, other kidney diseases, and healthy controls [6]. Therefore, the reported findings may not be useful in clinical practice where patients with active LN are mostly of class III/IV with active lesions in the kidney tissue [16]. Many patients with active LN have been observed to express certain molecular markers. However, there are no molecular markers that can be used to diagnose and prognose refractory LN. In the past, the authors reported that intra-renal *VEGF* expression can prognose patients with class III/IV LN [5]. When expression of the pro-angiogenic protein VEGF was lost, it was observed to be associated with loss of kidney function. This kind of biomarker is important for precisely prognosing such detrimental activity without the need for another invasive kidney biopsy. Using the data from this study, a pattern of molecular signatures can be developed to determine a poor prognosis for patients with severe forms of class III/IV LN. Correct interpretations of these patterns, such as increased *S100A8* and decreased *CLDN19* gene expressions in a patient with biopsy-proven class III/IV LN, may prove to be useful in predicting the patient’s therapeutic response before or during 6 to 12 months of treatment.

In this study, the authors detected five novel candidate biomarkers for diagnosing and prognosing refractory LN: *S100A8*, *ANXA13*, *CLDN19*, *FAM46B* and *COL8A1*. These biomarkers may be used as targets for future therapeutic regimens. There are some data for *S100A8* and *CLDN19*, but no data for *ANXA13*, *FAM46B* and *COL8A1*, indicating that additional investigations and studies are warranted. S100A8, also known as myeloid-related protein 8 (MRP-8), is expressed on monocytes and macrophages. It is a ligand of toll-like receptor 4 (TLR-4), which is involved in the development of autoreactive CD8 T-cells [17]. It is also a member of the damage-associated molecular patterns (DAMPs), which are one of the signatures indicative of the innate immune response. Previously, Frosch *et al.* [18] determined the expression of the S100A8 and S100A9 protein complex in macrophages infiltrating the kidneys in severe glomerulonephritis, including lupus glomerulonephritis. In this study, intra-renal *S100A8* expression was increased in the non-responders, which may indicate that monocytes and/or macrophages were infiltrating into the kidneys of the patients with active LN. The expression of *S100A8* on circulating blood leucocytes was increased in patients with LN [19] and may be a safer alternative to monitor LN.

Regarding CLDN19, it is a paracellular tight junction molecule of the thick-ascending limb of the nephron [20,21]. The authors detected a significant decrease in *CLDN19* during active LN. This biomarker may indicate a poor prognosis (*i.e.*, refractory to treatment and early loss of kidney function). This finding suggests a pivotal role for the tight junctions of the renal tubular cells in protecting the kidney from further injuries. The tight junction proteins can protect the polarity of the tubular cells and prevent the paracellular migration of substances [22]. A previous animal study showed expression of CLDN19 at the thick ascending limb with a function for renal magnesium reabsorption [23]. In human kidneys, expression of CLDN19 protein was located in the renal tubules, and decreased in the diseased kidney [24]. Hence, loss of the claudin expression can cause cellular dysfunction, and lead to tissue migration of toxic substances and inflammatory cells.

Aside from identifying biomarkers for diagnosing and prognosing refractory LN, this study also applied the unique technology of RNA microarrays to uncommonly used specimens such as kidney tissue. Tissue microarray analyses are widely performed in the field of cancer, and in a few reports on chronic kidney disease, glomerular diseases, or LN [25,26,27,28,29,30,31]. The limitation of this technology is the difficulty in performing microarray analysis on a tiny piece of kidney tissue [32,33]. In addition, the kidney tissue contains various types of cells and different portions of the nephron. Therefore, it is possible that the molecular signatures may be masked by the noise of the technique. However, this is highly unlikely because the molecular signatures for this type of LN were very strong and were validated by an independent set of kidney samples. Another limitation of this study is that gene expression analyses cannot determine the mechanism or pathway of the disease. It can be a non-response pathway or another activated pathway. Therefore, the biomarkers detected in this study will require more functional studies.

## 3. Experimental Section

### 3.1. Patients and Tissue Samples Collected

Sixty-seven patients who met the criteria for SLE and LN (Table 1 and Table 2) according to the revised criteria issued by the American College of Rheumatology were recruited into this study. Renal involvement was documented by having one of the following criteria: (1) total urinary protein level of more than 0.5 g/day; (2) increment of serum creatinine levels of more than 0.5 mg/dL during the one-month period of follow-up; or (3) presence of pyuria, hematuria, or urinary cast by microscopic examination [10]. The study was approved by the Ethics Committee for Human Research of the Faculty of Medicine, Chulalongkorn University (IRB No. 539/56) and written informed consent was obtained from all patients.

All LN patients with renal flare prior to initiating immunosuppressive therapy had a renal biopsy per protocol. All of the renal biopsy cores were collected and divided into two parts: one part was used for histology and the other part was used to obtain RNA. First, the frozen tissues were kept on ice and immediately transferred to a pathology laboratory for histological diagnosis. Second, the sections were transferred into RNAlater^®^ solution (Ambion Inc., Austin, TX, USA). The total RNA was extracted from the kidney tissue using an RNeasy Mini Kit (QIAGEN Inc. GmbH, Hilden, Germany) and measured with a NanoDrop ND-1000 UV-Vis Spectrophotometer (Thermo Fisher Scientific Inc., Wilmington, DE, USA).

All patients were initially treated with either mycophenolate mofetil or intravenous cyclophosphamide plus prednisone for six months and immunosuppression was maintained for three years. After six months of initial treatment, the following clinical criteria were used to assess the therapeutic responses: (1) stabilization or improvement in the renal function; (2) 50% decrease in hematuria to less than 10 RBC per high-power field; and (3) significant drop of proteinuria (50% decrease to less than 3 g/day if baseline was nephrotic range or less than 1 g/day if the baseline was non-nephrotic range) for at least three months [34]. After 12 months, kidney function was tested by the estimated GFR [35]. Early loss of kidney function was defined as an estimated GFR below 15 mL/min, or dialysis or kidney transplantation.

### 3.2. RNA Quality Control Assessment

To determine the RNA quality of the kidney tissue samples, the total RNA was tested for an RNA quality indicator (RQI) of more than 7.0 as well as the 28s/18s rRNA ratio, which was 1.7 to 2.0. The samples were then run on Illumina BeadChips and a quality control plot was acquired by GenomeStudio software (Illumina Inc., San Diego, CA, USA). Both sample-independent metrics controls (hybridization controls, low stringency, and biotin-high stringency plots) and sample-dependent metrics controls (negative control, gene intensity, and labeling-background plots) were assessed. These plots indicated that the performances of the BeadChips were satisfactory (data not shown). The microarray hybridizations on both BeadChips were evaluated for their concordance by replicating the same RNA samples on both BeadChips (correlation coefficient = 0.98).

The qualities of the extracted RNA were monitored by automatic electrophoresis using an Experion™ RNA StdSens Analysis Kit (Bio-Rad Laboratories, Hercules, CA, USA). The quality of the RNA was ranked according to the RNA RQI and scored from a range of 1 (degraded total RNA) to 10 (intact total RNA). Aside from the RQI scores, the 28s/18s rRNA ratio was also used. A ratio of 2.0 was considered to be of the best quality. In this study, only high-quality RNA samples were used for subsequent analysis (A260/A280 = 1.7–2.0, RQI ≥ 7, and 28s/18s rRNA ratio ≥ 1).

### 3.3. Gene Expression Microarray

The high-quality RNA samples (*n* = 23) were amplified onto the complementary RNA (cRNA) by an *in vitro* transcription technique using an Illumina^®^ TotalPrep RNA Amplification Kit (Applied Biosystems/Ambion, Austin, TX, USA). A starting template of 250 ng RNA of all total RNA samples was used for amplification. The obtained cDNA was transcribed onto the cRNA at 37 °C for 14 h and the cRNA was measured with a NanoDrop ND-1000 UV-Vis Spectrophotometer. Next, 750 ng of each cRNA sample was hybridized to Illumina^®^ HumanHT-12 Expression BeadChips (Illumina Inc.) and incubated at 58 °C for 18 h. After the incubation period was completed, all BeadChips were processed according to the manufacturer’s instructions for running the assay. Finally, the fluorescent signals were collected with an iScan machine and GenomeScan software (Illumina Inc.).

### 3.4. Microarray Data Analysis

GenomeStudio (Illumina Inc.) was used to examine the data quality and expression intensities of the BeadChips. Microarray analysis was performed using R-based Bioconductor packages (http://www.bioconductor.org) to determine the level of each gene expression signal. The DEGs from the non-responders and responders were analyzed and compared with a *t*-test to detect their possible impacts in predicting the therapeutic response. A value of *p* < 0.05 with an expression ratio of >1.3-fold was used to select the candidate genes. To identify the genes associated with the loss of the kidney function process, all LN patients were re-classified into patients whose kidney function was or was not lost within 12 months. To obtain a list of candidate biomarkers, not only the statistical test and expression ratio, but also the disease-related biological functions of the DEGs using functional annotation analysis were considered. Subsequently, both upregulated and downregulated genes for refractory LN and loss of kidney function were submitted to functional annotation analysis using the DAVID (http://david.abcc.ncifcrf.gov/) [36] and PANTHER (http://www.pantherdb.org/) [37] web-tools to assess the in-depth biological functions of the genes. Both services extracted the biological meaning of both sets of genes by retrieving their functional annotations from the KEGG (Kyoto Encyclopedia of Genes and Genomes) and GO (Gene Ontology) databases.

### 3.5. Validation of the Candidate Biomarkers

The candidate biomarkers were validated in a validation set (*n* = 44) using real-time PCR. In brief, RNA was reverse-transcribed onto the cDNA using a TaqMan^®^ Reverse Transcription Kit (Applied Biosystems, Carlsbad, CA, USA). The gene expressions of the candidate biomarkers were then quantified using a SYBR Green-based detection technique with an ABI Prism 7500 Real-Time PCR system. The sequences of the oligonucleotide primers used in this study can be found in Supplementary Table S7. The expression levels of the targeted genes were analyzed by the comparative CT method using 18s rRNA as the housekeeping gene.

### 3.6. Statistical Analysis of Real-Time PCR Validation

Statistical analysis was performed using SPSS software version 16 (SPSS Inc., Chicago, IL, USA). For continuous variables, data were expressed as mean ± SE. Differences between groups were analyzed by the Mann–Whitney *U*-test. Correlation coefficients were calculated by Spearman’s *ρ*-test. A receiver-operator characteristic curve analysis of the mRNA levels was used to determine the cutoff levels that maximized the combined sensitivity and specificity for the therapeutic resistance and loss of renal function within 12 months. The area under the curve was calculated, and the sensitivity and specificity at the selected cutoffs were determined. A value of *p* < 0.05 was considered statistically significant. Dot-plot graphs were created using GraphPad Prism version 4.03 (GraphPad Software Inc., La Jolla, CA, USA).

## 4. Conclusions

To conclude, the main purpose of this study was to identify biomarkers that can be used to diagnose and prognose refractory LN by applying the technology of genome-wide microarray analysis to the kidney tissue. The unprecedented molecular signatures measured by the quantitative real-time PCR proved to be correlated with refractory LN. Future functional studies of these molecules are needed to determine whether they can serve as both biomarkers and molecular targets for LN therapy.

## Supplementary Information

Supplementary materials can be found at http://www.mdpi.com/1422-0067/16/06/14276/s1.
