# Supplementary material for: Biomarkers for Refractory Lupus Nephritis: A Microarray Study of Kidney Tissue
Source: Int J Mol Sci. 2015 Jun 23;16(6):14276–90. doi: 10.3390/ijms160614276 (PMC4490552; doi:10.3390/ijms160614276)
Supplement: Supplementary file 1 [file ijms-16-14276-s001.zip › ijms-85476-Supplementary Information/ijms-85476-supplementary Table S5-S7.pdf]

Supplementary Information

Table S5. Comparisons of the expressions of the 8 validated genes in LN non-responder (NR; $N = 22$) and responder (R; $N = 22$) patients.

	**R**	**NR**	***p*-Value**
	Up-regulated genes		
ANXA13	−0.006 ± 0.75	1.65 ± 0.37	0.04
VCAM1	−0.007 ± 0.29	2.10 ± 1.19	0.09
VNN1	−0.0005 ± 0.36	0.97 ± 1.21	0.63
CXCR1	−0.001 ± 0.22	0.59 ± 0.30	0.24
S100A8	−0.07 ± 0.30	1.58 ± 0.50	0.02
	Down-regulated genes		
CLDN19	−0.005 ± 0.26	−0.87 ± 0.31	0.03
DDIT4	−0.0005 ± 0.28	−0.60 ± 0.31	0.11
FAM46B	−0.005 ± 0.62	−0.71 ± 0.28	0.02

Data shown as Mean ± SEM $\log_2$ fold change.

Table S6. Comparisons of the expressions of 4 validated genes in LN patients with loss of kidney function (L; $N = 13$) and preserved kidney function (P; $N = 31$).

	**L**	**P**	***p*-Value**
	Up-regulated genes		
ANXA13	1.61 ± 0.15	0.18 ± 0.80	0.34
COL8A1	0.99 ± 0.24	−0.004 ± 0.24	0.007
	Down-regulated genes		
SERPINA1	−0.22 ± 0.12	−0.007 ± 0.25	0.45
TRPV6	−0.06 ± 0.27	−0.004 ± 0.22	0.98

Data shown as Mean ± SEM $\log_2$ fold change.

Table S7. The sequences of oligonucleotide primers for real-time PCR validation.

TargetID	Oligonucleotides Sequence
ANXA13	Forward 5′-CAGGGTTTTGATGTGGATCGA-3′<br>Reverse 5′-TGCTTCATTGGTCCCCATTC-3′
VCAM1	Forward 5′-CCACAGTAAGGCAGGCTGTA-3′<br>Reverse 5′-GGTCACAGAGCCACCTTCTT-3′
VNN1	Forward 5′-GATCAGGGTGCGCATATTATTGT-3′<br>Reverse 5′-TTTACTTCAGGGTCTGGGATGTC-3′
CXCR1	Forward 5′-TGACTGGCAGATCCAGAGGTT-3′<br>Reverse 5′-ACTCAAAGTTTTCTGTCACTGATTCAG-3′
S100A8	Forward 5′-GAAGAAATTGCTAGAGACCGAGTG-3′<br>Reverse 5′-CGCCCATCTTTATCACCAGAA-3′
CLDN19	Forward 5′-TGGGTGGGCATCATTGC-3′<br>Reverse 5′-GTCGCCTGCGTAGGAAGACT-3′
DDIT4	Forward 5′-GGGTTCGCACACCCATTC-3′<br>Reverse 5′-GAGCGTAGAAGCCGCAGCTA-3′
FAM46B	Forward 5′-ACAGGTGAAGCGACTGGAC-3′<br>Reverse 5′-GTGCACATGTAGTCCCTGCT-3′
COL8A1	Forward 5′-AGCCCTTCCCCGATCCTCTC-3′<br>Reverse 5′-CACAACAGCTTCTTGAGCACG-3′
SERPINA1	Forward 5′-TCCGATAACTGGGGTGACCT-3′<br>Reverse 5′-GCATTGTCGATTCACTGTCCC-3′
TRPV6	Forward 5′-TGGAGCAAGTTCTGCAGATGG-3′<br>Reverse 5′-AGACTCCCAGATCCTCTTCTGCT-3′
